# Comprehensive Analysis of Key Parameters Determining Formation and Structural Properties of Sol–Gel‐Derived Nanoporous Polymers

**DOI:** 10.1002/smsc.202500460

**Published:** 2025-11-18

**Authors:** Abdurrahman Bilican, Priyanka Sharma, Glen J. Smales, Markus Leutzsch, Christophe Farès, Heike Ehmann, Armin Moser, Claudia Weidenthaler, Wolfgang Schmidt

**Affiliations:** ^1^ Department of Heterogeneous Catalysis Max‐Planck‐Institut für Kohlenforschung Kaiser‐Wilhelm‐Platz 1 45470 Mülheim an der Ruhr Germany; ^2^ Institute for Inorganic Chemistry Graz University of Technology Stremayrstraße 9/IV 8010 Graz Austria; ^3^ Department of Nuclear Magnetic Resonance Spectroscopy Max‐Planck‐Institut für Kohlenforschung Kaiser‐Wilhelm‐Platz 1 45470 Mülheim an der Ruhr Germany; ^4^ Anton Paar GmbH Anton‐Paar‐Str. 20 8054 Graz Austria

**Keywords:** in situ studies, reaction kinetics, resorcinol‐formaldehyde gels

## Abstract

This study presents a comprehensive investigation on the relationship between structure, synthesis parameters, and porous properties of sol–gel‐derived polymer gels. The formation of the porous gels is monitored with in situ small‐angle X‐ray scattering, in situ nuclear magnetic resonance spectroscopy (NMR), and NMR cryoporometry. The transition of the reaction solution to a solid gel is governed by the consumption of the phenolic monomer. Primary particle growth and nanopore formation proceed during this short time period and are completed when all resorcinol is consumed. The kinetics of these processes are temperature‐dependent and they are completed within 12 min at 120 °C and within 60 min at 80 °C. Extending the reaction time further results in enhanced cross‐linking of the polymer, as observed by solid‐state ^13^C NMR spectroscopy. Extended reaction time, i.e., higher degree of polymer cross‐linking, enhances pore stability and reduces gel shrinkage during drying, resulting in xerogels with larger pore volume, larger external surface area, and larger average pore sizes. This work rationalizes molecular‐scale transformation of polymers with macroscopic properties, thus providing a rational tool for tuning aerogel/xerogel performance through synthesis design.

## Graphical abstract

A comprehensive study shows that chemical composition of the gelation mixture determines the primary particles size and the size of pores they form. Reaction temperature and time define the degree of cross‐linking within the polymer and thus determine the mechanical strength of the gel. High mechanical strength results in low degree of shrinkage during thermal drying.

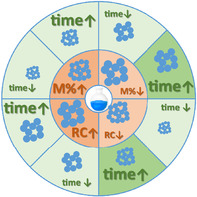

## 1 Introduction

The sol–gel synthesis of resorcinol‐formaldehyde (RF) gels and carbon aerogels/xerogels derived thereof are well‐established and extensively studied for the production of porous materials.^[^ ^1^, ^2^ ^]^ These materials have found application across diverse fields due to their tunable hierarchical porosity, allowing for the optimization of their structural and functional properties.^[^ ^3^, ^4^, ^5^ ^]^ Despite their potential, carbon gels face significant drawbacks related to extended synthesis times and often moderate reproducibility. Furthermore, solvent exchange and exceptional drying methods, such as supercritical or cryo‐drying, are often required to prevent porosity loss caused by capillary forces exerted during the drying process.^[^ ^6^ ^]^ These processes are thus time‐consuming and costly, adding to the complexity of carbon aerogel synthesis. This study revisits the long‐standing assertion that aerogels require extended reaction cycles of several days and that the reduction of capillary forces during drying can only be achieved through elaborated drying techniques or solvent exchange methods. Recent advancements, such as the “superfast synthesis”, have challenged these assumptions, demonstrating that carbon xerogels with tunable porosity can be synthesized in less than 5 h with high reproducibility.^[^ ^7^ ^]^ Central to this finding is a focus on the formation of the mesoporous polymeric gel precursor via the polycondensation of a phenolic or nucleophilic aromatic compound with an electrophilic aldehyde. The mechanism of RF gel formation has been extensively studied, particularly with time‐resolved analyses. The synthesis process is generally divided into two stages, i.e., gelation and aging. Gelation refers to the transition from a liquid reaction solution to a solid gel, which is typically marked by a rapid increase in viscosity. Pekala and Kong reported that after 200 min, nearly all the resorcinol in the reaction solution was consumed, while 40% of the formaldehyde remained unreacted. During this stage, small RF clusters form, with subsequent cross‐linking and polymerization driven by the remaining formaldehyde.^[^ ^8^ ^]^ Tamon et al. investigated the formation of these larger structures using in situ small‐angle X‐ray scattering (SAXS), revealing that while small clusters formed rapidly within hours, the development of fractal structures and a fully cross‐linked polymeric network could take several days at room temperature.^[^ ^9^ ^]^ By conducting similar SAXS experiments at elevated temperatures (70 °C), it was shown that the polymer framework was established within ≈300 min.^[^ ^10^ ^]^ The second stage, i.e., aging, is crucial for the mechanical stability and porosity of the gel. Studies by Despetis et al. demonstrated that the mechanical stiffness (shear modulus, G) of the gel could be enhanced by aging in acidic solutions, which promotes the formation of methylene bridges within the polymer network.^[^ ^11^ ^]^ Wiener et al. showed that the synthesis of RF gels can be shortened to 1 day by heating to 90 °C during gelation and aging, resulting in slightly smaller particle and pore sizes, which can be counterbalanced by adjusting the reaction solution. Additionally, solvent exchange can be optimized experimentally or omitted to save time, although this may decrease pore sizes due to higher capillary forces.^[^ ^12^ ^]^ While these studies have elucidated the factors contributing to the long gelation and aging times of mesoporous RF gels, questions remain with respect to the mechanisms by which elevated temperatures, as used in the superfast synthesis, accelerate the formation of stable RF gels to reaction times as short as 1 h and, at the same time, maintaining porous properties like gels synthesized at 80 °C for 24 h.^[^ ^7^ ^]^ This study addresses these questions by investigating the effect of elevated synthesis temperatures on the kinetics of porous network formation, polymer cross‐linking, and the resulting pore characteristics. In the past, related information was spread out in numerous publications, where a wide range of synthesis parameters were used, making comparison of systems quite challenging. Here, now a set of highly complementary analysis methods was used, namely in situ SAXS, in situ ^1^H NNMR, ^13^C solid‐state nuclear magnetic resonance spectroscopy (NMR), NMR cryoporometry, and cryogenic N_2_ Sorption analysis on identical reaction systems. This unique combination of methods provided clear data‐based insights into the formation of the RF gels on the molecular level and allowed linking that information to structural and materials properties of the gels.

## 2 Results and Discussion

**Figure 1:**
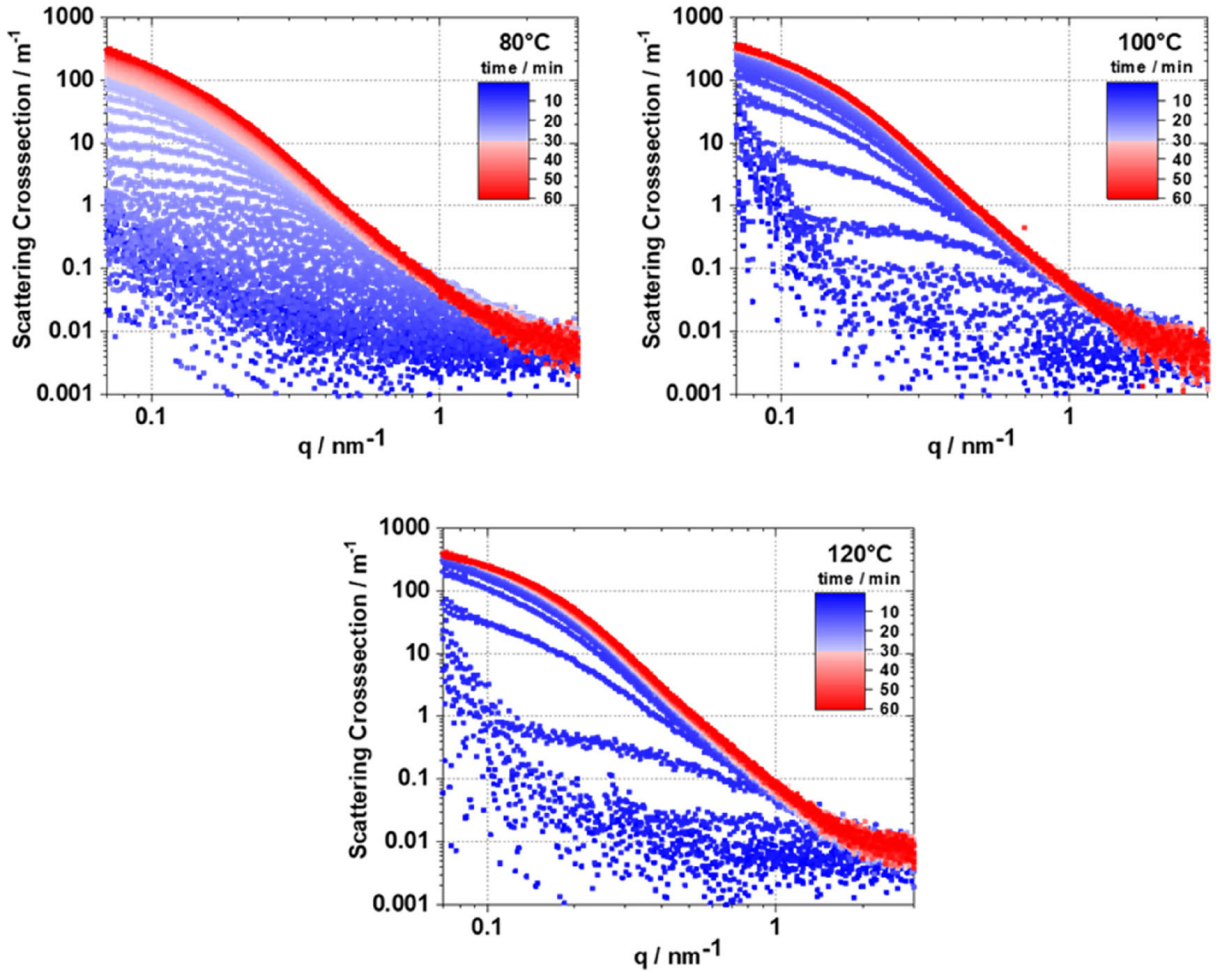
In situ SAXS curves obtained during gelation of RF gels in reaction solutions at 80, 100, and 120 °C using an RC/M% ratio of 750/30.

In situ SAXS experiments provided direct insight into the kinetics of pore formation during the synthesis of RF gels at 80, 100, and 120 °C (**Figure** **1**). At early reaction times, scattering intensities were low across the entire q range, indicating minimal structural contrast within the system. As the reaction progressed, a pronounced increase in intensity, particularly between 0.07 and 1 nm^−^^1^, was observed, reflecting the formation and growth of electron density fluctuations associated with the developing porous network. Temperature is seen to have a strong effect on the rate of this structural transformation. At 80 °C, scattering curves evolved gradually over ≈50 min before reaching a stable state with no further significant changes. At 100 and 120 °C, the formation of the final structure occurs more rapidly, reaching a plateau within ≈20 and 10 min, respectively. This trend indicates that the formation rate of the pore network is highly temperature‐dependent and can be significantly accelerated under hydrothermal conditions.

**Figure 2:**
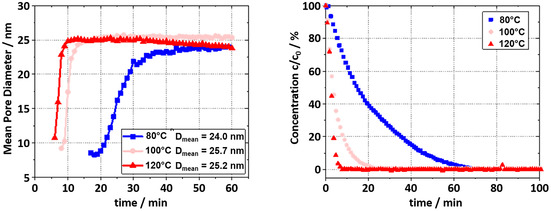
Mean pore sizes derived from SAXS over time (left) and concentration decay of resorcinol inside the reaction solution during RF gel formation measured by ^1^H NMR (right).

To quantify these observed changes, the SAXS data were fitted using the McSAS3, employing a spherical pore model. This allowed for the extraction of time‐resolved size distributions without assuming a specific distribution shape. The resulting mean pore sizes are shown in **Figure** **2** (left). All samples exhibited similar growth trajectories, eventually plateauing at diameters of ≈24–26 nm. Notably, the sample synthesized at 120 °C showed a slight decrease in mean pore size toward the later stages of the in situ experiment. This shrinkage is thought to be caused by solvent evaporation, possibly due to poor sealing of the sample environment at elevated temperature. To better understand the gelation process, Figure 2 (right) shows the decrease of resorcinol monomer concentration in the reaction solution, as determined by ^1^H NMR (see Supporting Information for details), correlated with the convergence of pore size observed by in situ SAXS.

As shown in **Table** **1**, the complete consumption of resorcinol is nearly synchronous with the pore size alignment derived from SAXS. This evidence supports the hypothesis that the formation of the porous structure coincides with the complete consumption of resorcinol monomers within the reaction solution. Although radius of gyration (*R*_G_) analysis was not performed, the overall evolution of the SAXS curves qualitatively matches the gelation behavior described by Tamon et al., where increasing low‐q intensity and eventual stabilization were linked to network formation.^[^ ^9^ ^]^ However, classical *R*_G_ analysis, derived from Guinier fits, yields only a single averaged length scale and cannot resolve polydispersity. In contrast, the spherical pore model used in McSAS provides a more detailed picture by capturing the full size distribution, offering greater insight into the structural evolution during gelation (see example fits and distributions in Figure S4–S6, Supporting Information, with the complete dataset available on zenodo). Prolonged test over 3 h at 100 °C, as shown in Figure S7, Supporting Information, reveals that the mean particle sizes remained unchanged.

**Table 1:** Time required for full consumption of resorcinol (NMR) and time to achieve pore size convergence (NMR) upon formation of RF gels (RC/M% = 750/30), as well as mean pore diameters of the RF gels, as determined by SAXS.

*T* [°C]	NMR [min]	SAXS [min]	D_mean_ [nm]
80	66	60	24.0
100	24	21	25.7
120	8	12	25.2

**Figure 3:**
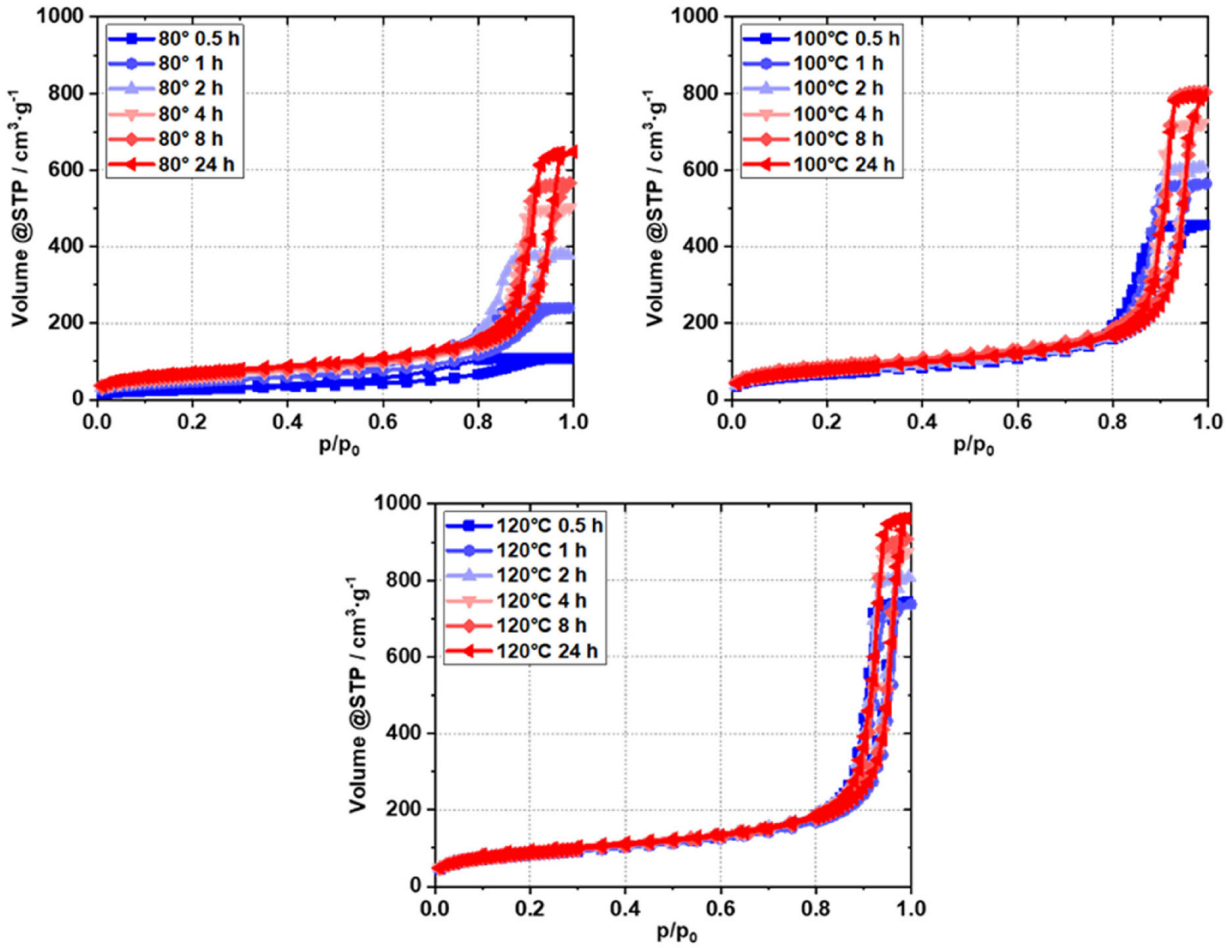
Nitrogen sorption isotherms of dried xerogels obtained using different reaction times (0.5–24 h) and temperatures (80, 100, and 120 °C) during formation of the pristine RF gels (RC/M% = 750/30).

Having determined by in situ SAXS that for an RF gel with a reaction solution composition of RC/M% 750/30, the formation of the porous framework at a temperature between 80 and 120 °C takes between 12 and 60 min, the question now arises as to how the temperature used affects the resulting pore size of the xerogel obtained after an ambient drying process. The nitrogen sorption isotherms of dried xerogels synthesized at 80, 100, and 120 °C and obtained after reaction times between 0.5 and 24 h are shown in **Figure** **3**. The isotherms illustrate the pronounced dependence of time and temperature. Evidently, elevated temperatures consistently result in enhanced porosity at given reaction times.

The isotherms of the samples at 80 °C alone show a significant change in porous properties over the reaction time studied. According to IUPAC, the isotherms are all of type IV, but as the synthesis time progresses (from blue to red), a greater amount of nitrogen is adsorbed, indicating an increase in pore volume. The samples also have different shapes in their hysteresis loops. The 0.5, 1, and 2 h samples show H2‐type hysteresis loops.

The mean pore diameters D_mean_ of these samples, as calculated by the Barret–Joyner–Halenda (BJH) method, lie between 5.9 and 11.4 nm (**Table** **2**). The pore diameters are thus above the critical neck width of 5–6 nm, below which cavitation effects are known to proceed.^[^ ^13^ ^]^ Starting from a synthesis time of 4 h, the pore size gradually increases up to a value of 18.6 nm at 24 h, and the hysteresis loops can be classified as H1 type. As the reaction time increases, not only does the pore volume V_pore_ increase steadily, but also the specific external surface area S_ext_, as shown in Table 2. All textural parameters (pore volumes, specific surface areas, and pore diameters) increase with time. For the samples synthesized at 100 °C, the typical type IV isotherms show H1 hysteresis loops already after 0.5 h. The above‐mentioned textural parameters, as listed in Table 2, for these samples are all higher than those for the sample obtained at 80 °C after ≈2 h. The relative increase in pore volume, external specific surface area, and pore diameter at the respective synthesis times is lower than for the 80 °C sample, although the absolute values are significantly higher at given reaction times. This trend continues consistently for the samples obtained at 120 °C. The textural parameters get maximized after 24 h at 120 °C.

**Table 2:** Porous properties of dried xerogels derived from isotherms in Figure 3 for samples synthesized at 80, 100, and 120 °C at synthesis times of 0.5–24 h.

T [°C]	*t* [h]	*V*_pore_ [cm^3^ g^−1^]	*V*_meso_ [cm^3^ g^−1^]	*V*_mic_ [cm^3^ g^−1^]	*S*_BET_ [m^2^ g^−1^]	*S*_ext_ [m^2^ g^−1^]	D_mean_ a) [nm]
80	0.5	0.167	0.159	0.008	96	78	5.9
	1	0.372	0.358	0.014	170	137	8.2
	2	0.573	0.554	0.019	217	172	11.4
	4	0.776	0.764	0.012	214	185	15.5
	8	0.877	0.855	0.022	234	182	17.0
	24	1.004	0.98	0.024	251	195	18.6
100	0.5	0.708	0.695	0.014	241	206	12.6
	1	0.875	0.858	0.017	264	221	14.6
	2	0.939	0.924	0.015	259	218	16.3
	4	1.120	1.098	0.023	293	237	18.0
	8	1.245	1.221	0.025	303	242	19.8
	24	1.234	1.214	0.019	280	231	20.2
120	0.5	1.153	1.129	0.024	297	239	18.7
	1	1.143	1.119	0.024	296	236	19.5
	2	1.254	1.229	0.025	309	248	20.0
	4	1.354	1.328	0.026	310	247	21.9
	8	1.408	1.383	0.025	311	249	22.7
	24	1.492	1.466	0.026	317	255	23.5

a) Calculated with the BJH method.

**Figure 4:**
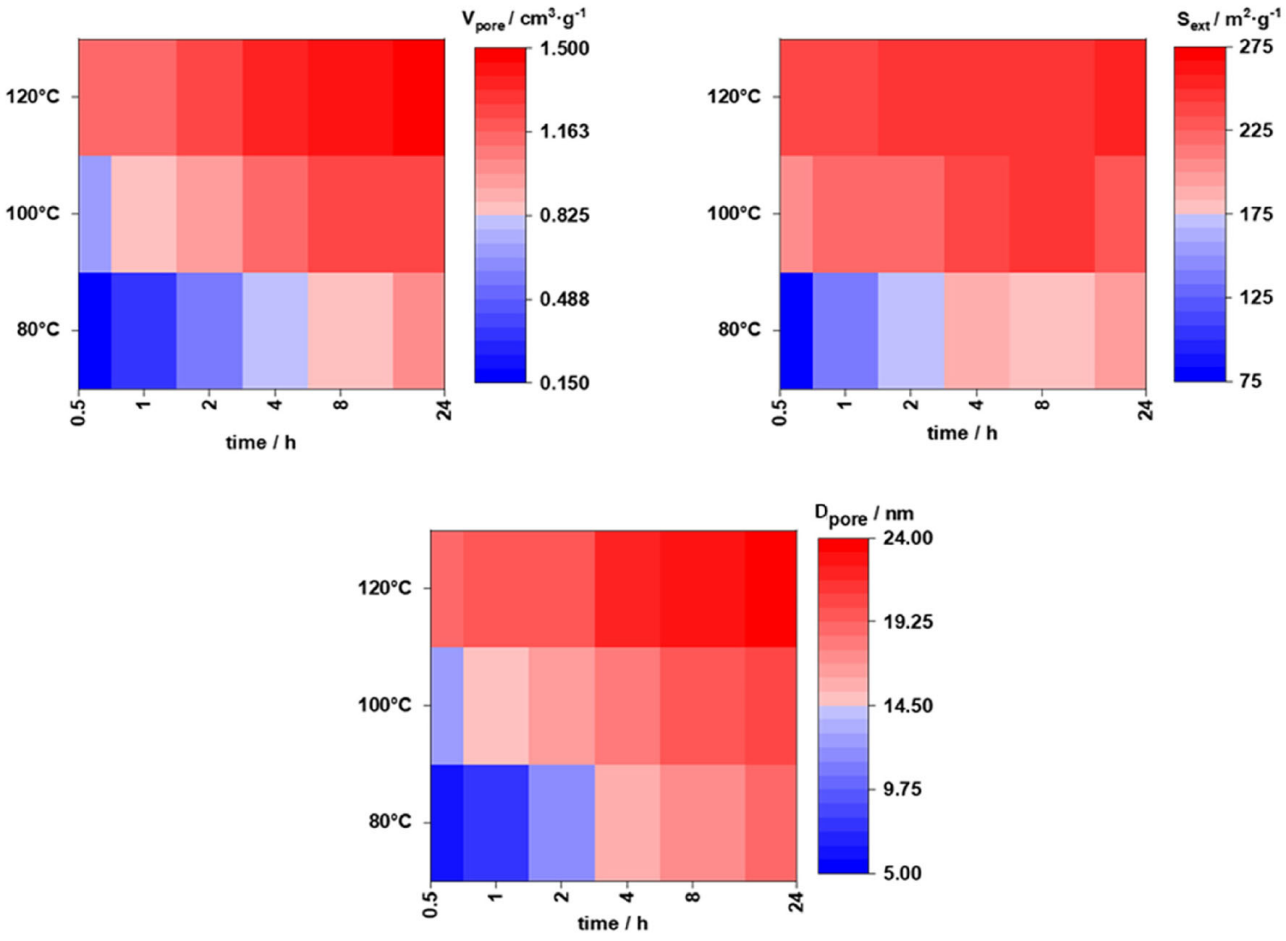
Time‐temperature matrices of total pore volume V_pore_, specific external surface area S_ext_, and pore diameter D_pore_ of xerogels obtained via drying of RF gels obtained at different temperatures and reaction times.

**Figure** **4** shows biaxial matrices for total pore volumes, external surface areas, and average pore sizes with synthesis time on the abscissa and reaction temperature on the ordinate. All matrices indicate that porous properties can be maximized with longer synthesis time and higher temperature. As illustrated in Figure 4 and Table 2, the pore volumes, specific surface areas, and pore diameters increased through the hydrothermal synthesis process. A maximized porosity is achieved after 24 h at 120 °C.

**Figure 5:**
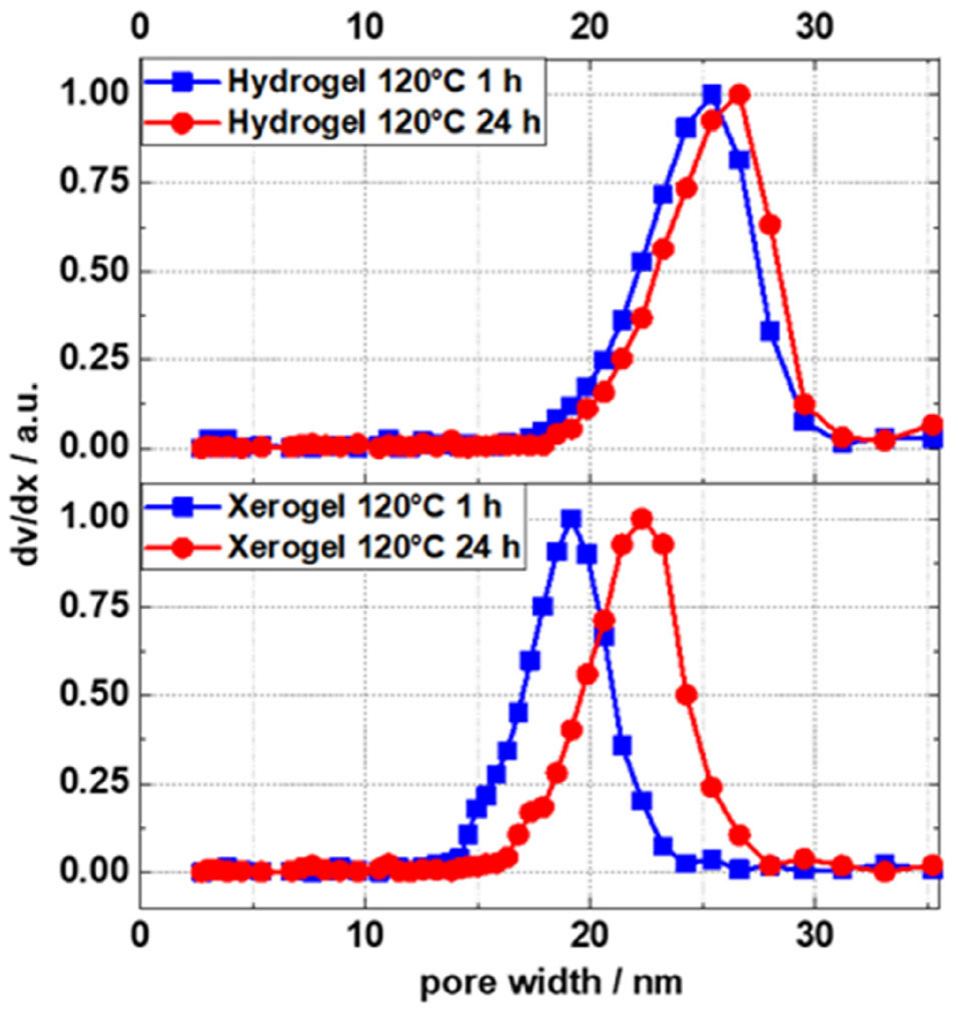
Pore size distribution of hydrogels (RC/M% = 750/30) obtained after 1 and 24 h (top) and those of the respective xerogels (bottom) after 24 h drying at 80 °C, as derived from melting curves measured by ^1^H NMR.

In situ SAXS and NMR proved that the mean pore sizes and resorcinol consumption remained unaltered after relatively short time periods in the range of ≈10 min (120 °C) to 60 min (80 °C), with pore sizes reaching the range of 24–25 nm for samples. Thus, extended reaction times apparently do not result in larger pores or pore structures in the wet RF gels. Nevertheless, the data shown in Table 2 and Figure 4 show that the textural parameters of the dried xerogels, including pore diameters, varied significantly with time and reaction temperature. To resolve the cause for these contradicting observations, focus was laid on the processes proceeding during aging and upon drying of the RF gels. To evaluate the relative shrinkage of the pores upon drying of the RF gels investigated here, NMR cryoporometry was applied on pristine wet RF gels, denoted as hydrogels, and on dried (24 h at 80 °C) xerogels. The method of NMR cryoporometry is explained briefly in the experimental section and in more detail in the supporting information. Basically, water gets frozen in the pores of the sample by decreasing the temperature well below 273 K. With increasing temperature, water melts within nanopores at temperatures below the melting temperature of bulk water. The observed melting temperature depression is then translated into pore diameters (see Supporting Information). Liquid water fractions are determined from ^1^H NMR spectra measured at defined temperatures upon heating, allowing calculation of pore size distributions as shown in **Figure** **5**. The pore size distributions of wet RF hydrogels obtained directly after synthesis, without exposure to drying‐induced shrinkage, revealed striking similarities irrespective of reaction time. As shown in Figure 5 (top) and **Table** **3**, the mean pore sizes for hydrogels synthesized at 120 °C for 1 h and 24 h were 24.1 and 25.3 nm, respectively, aligning closely with pore size values derived from SAXS data. In contrast, mean pore sizes of the dried xerogels (Figure 5 (bottom) and Table 3) strongly differ from these values, highlighting the impact of synthesis time and aging on shrinkage behavior. Xerogels obtained by drying hydrogels synthesized for 1 h exhibited a mean pore diameter of 18.1 nm, while those obtained from hydrogels synthesized for 24 h showed a significantly larger mean pore diameter of 21.4 nm. Upon drying of both hydrogels, a shrinkage of the pores is observed. However, for the one obtained after 1 h, 25% relative shrinkage is observed, whereas only 15% relative shrinkage is found for that obtained after 24 h. This reduction in relative shrinkage demonstrates the effect of extended hydrothermal treatment, i.e., increased structural integrity upon drying, well in accord with the work of Despetis et al., reporting that extended aging results in higher mechanical strength of RF gels.^[^ ^11^ ^]^

**Table 3:** Mean pore sizes of RF xerogels derived from N_2_ sorption (D_Xerogel_, BJH) and of hydrogels (x_Hydrogel NMR_) and xerogels (x_Xerogel NMR_) derived from NMR cryoporometry and their relative pore size reduction ε after drying.

Sample	D_Xerogel BJH_ [nm]	x_Hydrogel NMR_ [nm]	x_Xerogel NMR_ [nm]	*ε* [%]
120 °C 1 h	19.5	24.1	18.1	25
120 °C 24 h	23.5	25.3	21.4	15

**Figure 6:**
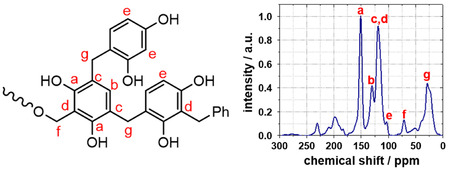
Representative molecular substructure of cross‐linked RF xerogel (left) and ^13^C CP‐MAS NMR spectrum of a RF xerogel (right).

For determining the origin of the stability of the RF networks, ^13^C CP‐MAS NMR provided information on the type of chemical bonds within the polymer network. As resorcinol reacts with formaldehyde, a porous cross‐linked phenolic polymer forms, exemplified by the representative substructure shown in **Figure** **6** (left). The condensation reaction of base‐catalyzed resorcinol and formaldehyde gels involves the formation of a quinone methide from resorcinol and subsequently, a Michael addition between the quinone methide and a resorcinol anion.^[^ ^14^ ^]^

This sequence of events leads to the propagation of the chain reaction, resulting in the formation of a cross‐linked polymer through methylene and methylene ether bridges. The two hydroxyl groups of resorcinol have an electron‐donating effect and direct the reaction with formaldehyde to the *ortho* and *para* positions. Thus, a C—C linkage is formed predominantly on positions 2, 4, and 6 of the resorcinol ring, whereas a C—C bond is thermodynamically most favored on position 2,4.^[^ ^14^ ^]^ The ^13^C NMR spectra of an RF‐xerogel are displayed in Figure 6 (right). The resonances provide information on C—C bonds and their position within the RF framework. A fraction of the ^13^C resonances can be assigned to different aromatic carbons, as shown in Figure 6. Aromatic carbons attached to a hydroxyl group (***C***_**aro**_ **‐OH**) can be assigned to resonances between 155 and 157 ppm, labeled as position a in Figure 6. Unsubstituted aromatic carbons (***C***_**aro**_ **‐H**) give resonances between 130 and 131 ppm (b, resorcinol position 5) and 103 and 105 ppm (e, resorcinol position 2, 4, and 6). This provides information about positions available for C—C linkages, with linkage at position e being thermodynamically more favored than at position b. Resonances of aromatic carbons which are connected to methylene bridges (***C***_**aro**_ **‐CH**_**2**_) are observed at 123–128 ppm (c, resorcinol position 4 and 6) and 112–117 ppm (d, resorcinol position 2) and give information about the occurrence of C—C linkages formed on the aromatic ring. Resonances from carbon atoms within a methylene bridge connected to two aromatic carbons (**C**_**aro**_ **‐** ***C*** **H**_**2**_ **‐C**_**aro**_ **)** are seen at position g at 25–33 ppm. Additional resonances of carbons connected to methylene ether (**C**_**aro**_ **‐CH**_**2**_ **‐O‐CH**_**2**_ **)** on position f are located at around 55–72 ppm (**Table** **4**).

**Table 4:** Assignment of ^13^C resonances to carbons within the polymer network.

Group	Position	Chemical shift [ppm]
*C*_aro_‐OH	a	155–157
*C*_aro_‐H	b	130–131
*C*_aro_‐CH_2_	c	123–128
*C*_aro_‐CH_2_	d	112–117
*C*_aro_‐H	e	103–105
C_aro_‐*C*H_2_‐O‐CH_2_	f	55–72
C_aro_‐*C*H_2_‐ C_aro_	g	25–33

For the analysis, special attention was paid to the resonance intensities at positions d and e, as the increase in intensities of resonances c and d (C—C linkage position) and the decrease in resonance **e** (free cross‐linking position) indicate the progress of the polymerization reaction. By careful peak deconvolution, the degree of cross‐linking could be quantified over the course of the reaction under different temperature conditions.

**Figure 7:**
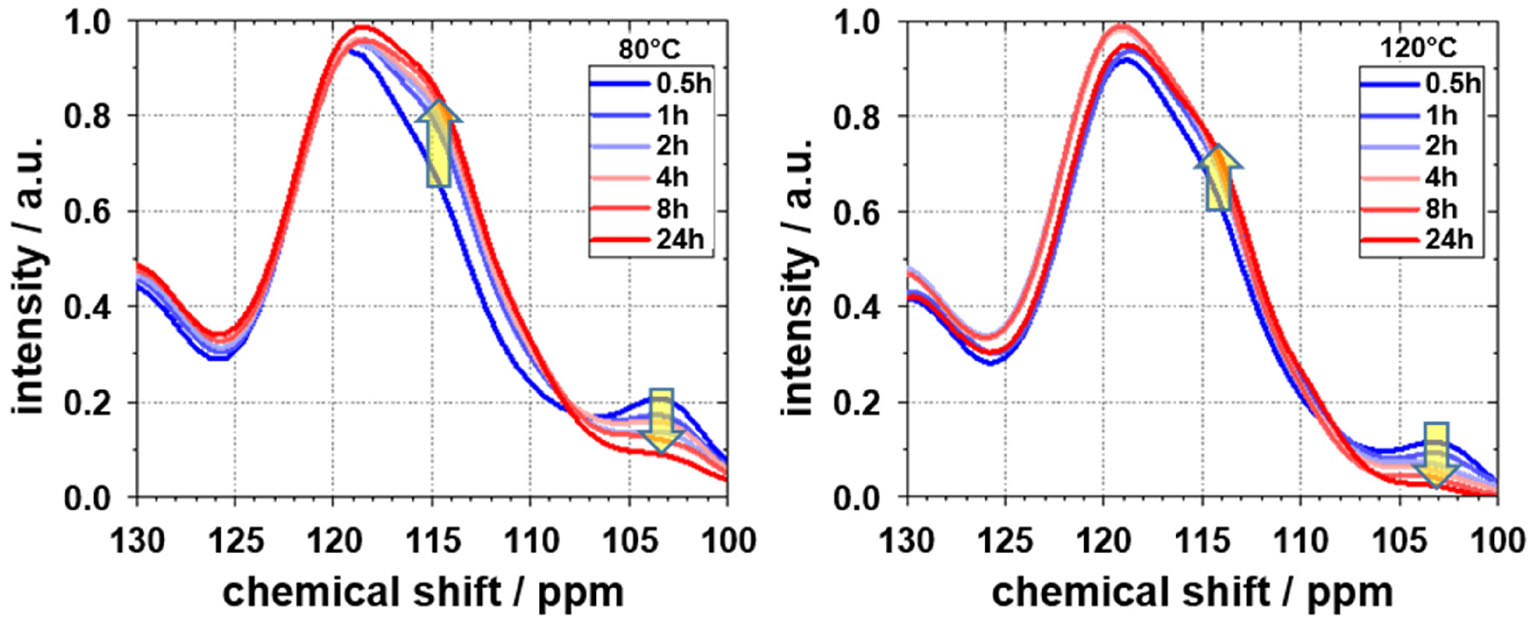
^13^C CP‐MAS NMR spectra of an RF gel synthesized at 80 °C (left) and 120 °C (right) at times between 0.5 h (blue) and 24 h (red).

In **Figure** **7**, the magnified NMR spectra of RF gels, synthesized at 80 °C (on the left) and at 120 °C (on the right), are presented. The ***C***_**aro**_ **‐H** signal (103–105 ppm) at position e, i.e., the number of free substitution positions available for cross‐linking, decreases with increasing synthesis time. Conversely, the ***C***_**aro**_ **‐CH**_**2**_ signal (112‐117 ppm) of carbon on position d increases in intensity, as additional methylene bridges are formed. The *C*_aro_‐CH_2_ resonances (d) for the samples obtained after 24 h exhibited the highest absolute intensities (see Table S1 and S2, Supporting Information), and their integrated intensities were designated as I_0_. Relating the intensities (I) of the respective resonances (d) of the other samples to this I_0_ allows for a quantitative assessment of the effect of time and temperature on the degree of cross‐linking. Higher concentration of CH_2_ methylene bridges within the polymer framework results from enhanced cross‐linking, as indicated by higher I/I_0_ values.

**Figure 8:**
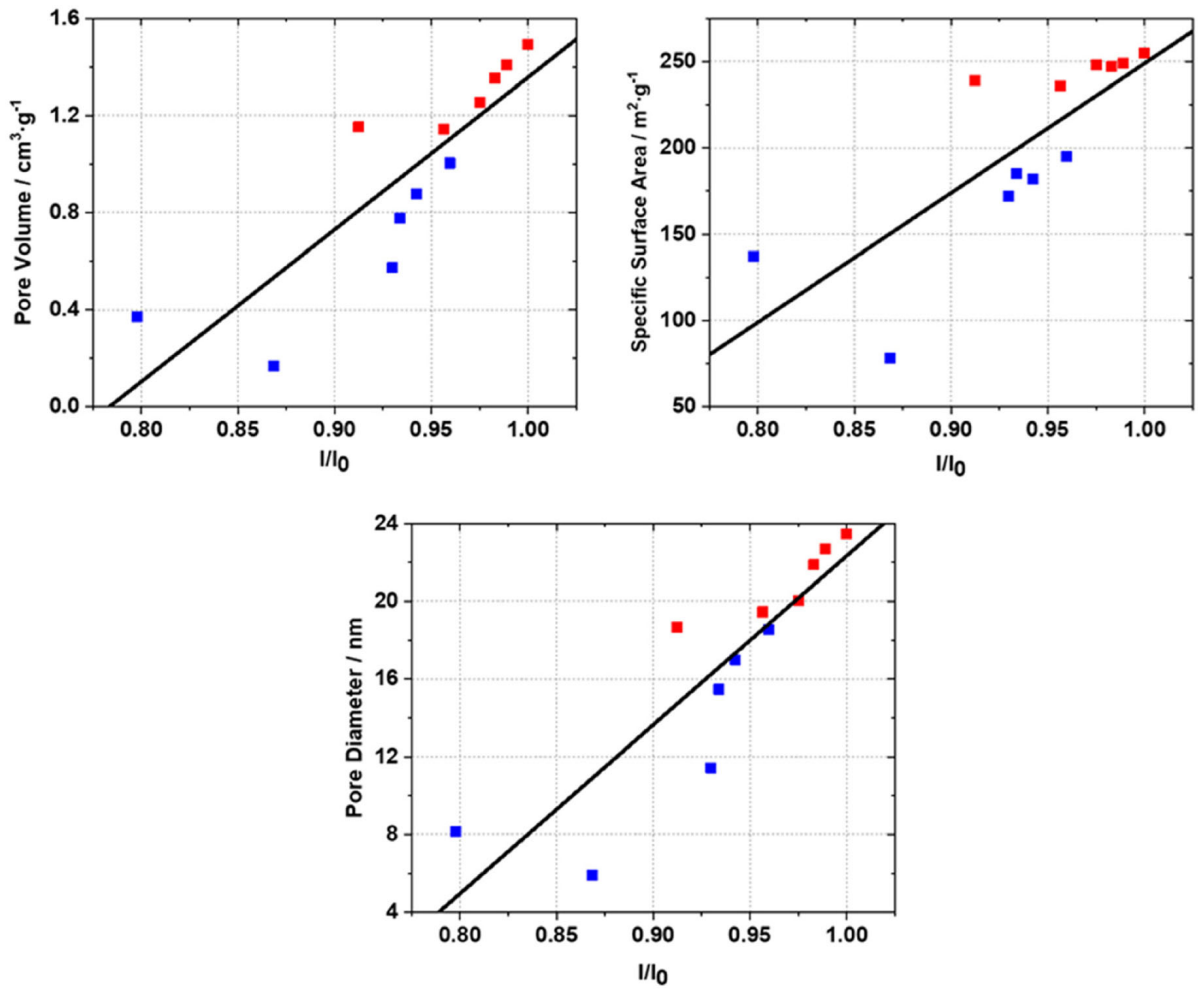
Relation between I/I_0_ of C_aro_‐CH_2_ resonances and pore volumes, specific surface areas, and pore diameters of RF xerogels synthesized at 80 °C (blue) and 120 °C (red).

The I/I_0_ ratios increase more or less linearly with time and temperature. Thus, enhanced cross‐linking, i.e., enhanced stability of the polymer, and the enhanced porous properties of the samples correlate. In **Figure** **8**, the pore volume V_pore_, specific external surface area S_ext_, and the mean pore diameter D_mean_ (Table 2) for samples obtained at 80 °C (blue) and 120 °C (red) are plotted versus I/I_0_ of the ***C***_**aro**_ **‐CH**_**2**_ resonances (d). The plots reveal a strong correlation between these parameters and the relative increase in methylene bridge signal (increase in I/I_0_). This observation strongly fosters the hypothesis that an increase in time and in temperature results in an increase in pore volume, in specific surface area, and in pore volume. The increase in the number of C—CH_2_—C bridges, i.e., higher cross‐linking, within the polymer network leads to higher mechanical strength of the porous polymer and thus to higher resistance against drying‐induced capillary pressure. Higher synthesis temperatures and prolonged synthesis times effectively maximize cross‐linking within an RF gel. In this way, pore size shrinkage and loss of porosity can be minimized.

## 3 Conclusion

This study provides a comprehensive analysis of the impact of reaction time and temperature on the physicochemical properties of sol–gel‐based porous polymer‐derived xerogels. Using in situ SAXS and in situ NMR measurements, the formation of porous structures, transitioning from a reaction solution to a solid gel, was demonstrated to be completed with the consumption of the resorcinol monomer. Furthermore, it was shown that for a given RC/M% ratio, the average pore size within the particle network, as determined from SAXS data, converges to a value of 25 ± 1 nm, irrespective of the reaction temperature. At 80 °C, this convergence occurs after approximately one hour, while at 100 and 120 °C, it is reached after 21 and 12 min, respectively. Additional NMR cryoporometry measurements revealed that hydrogels synthesized at 120 °C, with reaction times of 1 and 24 h, exhibit mean pore sizes of 24.1 and 25.3 nm, respectively, closely matching the mean pore size of 25 nm determined by SAXS. Pore size distributions of these polymers before and after drying showed that longer reaction times reduce the relative shrinkage from hydrogel to xerogel from 25% to 15%. This increase in pore diameter with prolonged reaction time was further confirmed through nitrogen sorption measurements. With increasing reaction time and temperature, a continuous rise in pore volume, specific external surface area, and average pore diameter was observed. These findings support the hypothesis that the development of porous properties is completed within minutes to hours, driven by the consumption of resorcinol. The porous properties present after ambient gel drying are primarily determined by the degree of cross‐linking within the polymer network. The degree of cross‐linking was relatively quantified across samples using ^13^C CP‐MAS NMR spectroscopy by correlating the intensity of cross‐linking‐related C_aro_‐CH_2_ resonances. The resulting linear correlation indicated that increasing relative intensities of cross‐linking‐related resonances correspond to increases in pore volume, external surface area, and average pore diameter. Finally, the study confirmed the relationship between enhanced mechanical properties and extended aging time previously described in literature, which has been attributed to cross‐linking in RF gels.^[^ ^11^ ^]^ It additionally quantitatively demonstrates the influence of aging time and temperature on the degree of cross‐linking and the resulting porous properties of the polymer network and draws an implied picture of chemical structure and porous properties relationship.

## 4 Experimental Section

4.1

4.1.1

##### Synthesis

Resorcinol (ACS reagent ≥ 99%, Sigma–Aldrich) was dissolved in a 37 wt% aqueous formaldehyde solution (ACS reagent, 36.5–38.0%, stab. with 10–15% methanol) at a molar resorcinol/formaldehyde ratio (R/F) of 1:2. An appropriate amount of water was then added to give a mass ratio M% of 30%.

(1) $$M\%=\frac{m_{\text{Resorcinol}}+m_{\text{Formaldehyde}}}{m_{\text{total}}}$$

For the synthesis of RF hydrogels with different porous properties, varying amounts of 0.05 m aqueous solution of Na_2_CO_3_ (Titripur , Merck KGaA Germany) were used to obtain the required resorcinol/sodium carbonate ratios (RC). The RC values were varied at 500, 750, and 1000.

(2) $$RC=\frac{n_{\text{Resorcinol}}}{n_{{\text{Na}}_{2}{\text{CO}}_{3}}}$$

The solutions were stirred briefly and then poured into Teflon liners, which were placed in steel autoclaves. The autoclaves were then placed in a heating block at 120 °C for defined reaction times ranging from 0.5 to 24 h. To obtain RF xerogels, the RF hydrogels were then removed from the autoclaves and dried at 80 °C for 24 h.

The synthesis parameters were adopted from previous experiments.^[^ ^7^, ^15^ ^]^ They were chosen to allow for representative experiments within a reasonable time and with concentrations enabling consistent data acquisition with the different characterization methods used.

##### In Situ SAXS

SAXS analysis was performed on a SAXS Point 2.0 instrument (Anton Paar) with point‐collimation of Cu Kα X‐Ray radiation (*λ* = 0.154 nm). An RF reaction solution was prepared as described elsewhere.^[^ ^7^ ^]^ Small droplets of this solution were held between Kapton windows using the PasteCell HS provided by Anton Paar. The in situ measurements were performed at 80, 100, and 120 °C using the Anton Paar temperature‐controlled stage (TCS). Samples were placed within the sample stage chamber and heated rapidly to the desired temperature while collecting data. Scattering data were collected on an Eiger 1M detector (Dectris, Switzerland) positioned 1064 mm from the sample. The collected data were then processed using standardized data correction procedures within the DAWN software package.^[^ ^16^, ^17^ ^]^ The corrected data were subsequently fitted using the McSAS3 software package, based on a Monte Carlo algorithm, to obtain form‐free size distributions, using a spherical model.^[^ ^18^ ^]^ Example fits of the SAXS data can be found in the Supporting Information, however, full datasets are available on zenodo (10.5281/zenodo.15130599).

##### N_*2*_ *Sorption*

For N_2_ sorption analyses at liquid nitrogen temperature, the RF xerogels were degassed under vacuum at 80 °C for 12 h prior to measurements on a Micromeritics 3Flex instrument. Data analysis was performed with the MicroActive software package (Micromeritics), applying the Brunauer–Emmett–Teller (BET) model for calculating apparent specific surface areas (S_BET_) according to the Rouquerol criteria.^[^ ^19^, ^20^ ^]^ The t‐plot method, using the Harkins and Jura thickness equation, was employed to calculate the micropore volume (V_mic_) and specific external surface area (S_ext_).^[^ ^21^, ^22^ ^]^ Pore diameters (D_pore_) in the meso‐ and macropore range were determined using the BJH method from desorption branches of the isotherms.^[^ ^23^ ^]^ Full datasets are available on zenodo (10.5281/zenodo.15130599).

##### In Situ ^*1*^ *H NMR Spectroscopy (Liquid State)*

The synthesis was followed for the RC of 750 and a fixed M% of 30. The reaction progress was investigated at three different temperatures, namely 80, 100, and 120 °C. After the reaction solutions were freshly prepared at room temperature, the samples were transferred to the NMR probe preheated to the respective temperature. Directly after insertion, single scan ^1^H NMR spectra were acquired on a Bruker Avance III 300WB spectrometer equipped with a 5‐mm BBFO probe with z‐gradient until the peaks of resorcinol from the solution phase fully vanished. The data were then imported into Mestrelab MNOVA 15.0.0 with the *Reaction Monitoring plugin* and processed therein (Fourier transformation, phasing, baseline correction, integration). To obtain the reaction profiles, the triplet signal at round 6.5–6.6 ppm was integrated, and the first acquired spectrum was used as concentration reference *t* = 0. Full datasets are available on zenodo (10.5281/zenodo.15130599).

##### NMR Cryoporometry

The coarse gel particles were then subjected to a grinding process with a minimal quantity of mQ water, resulting in a wet powder. Subsequently, the powder was immersed in mQ water for a period of 24 h, with the objective of achieving a fine dispersion. This dispersion was then transferred into 5‐mm screw cap NMR tubes. Next, cryoporometry measurements were conducted on a Bruker AVANCE III HD 400 spectrometer equipped with a 5 mm BBFO probe with z‐gradient and a Bruker BCU II cooling unit. Temperatures ranging from 283 to 243 K were precisely calibrated using the methanol chemical shift separation method (sample 4% methanol in MeOD) as automated in the Bruker Topspin AU program *calctemp*. Melting curves were obtained by freezing the in‐pore liquid until the NMR signal completely vanished and then heating the sample in successive temperature steps while collecting NMR data at each temperature until complete liquefaction (melting) of the in‐pore and bulk liquid was reached. At each temperature, the sample was maintained for 10 min to ensure temperature stability. Following this, the probe was automatically tuned and matched. A *T*_2_‐filter (Bruker sequence: *cpmg1d*, using a single spin echo with delay 2*d20 = 2 ms) was implemented to suppress residual signal from the solid. These acquisition parameters were previously described by Rotterau et al.^[^ ^24^ ^]^ The acquired NMR data were imported into Mestrelab MNOVA 15.0.0 with the *Reaction Monitoring* plugin and processed therein (Fourier transformation, phasing, baseline correction, integration). Generally, only a zero‐order phasing was used, and the baseline was corrected using a zero‐order polynomial. The signal of the water was integrated ± 15 ppm around the maximum peak. For the evaluation of the pore size distribution, the Gibbs‐Thomson temperature decrease Δ*T*_m_ was used as described in the Supporting Information.^[^ ^25^, ^26^ ^]^ Pore size shrinkage was determined as described elsewhere.^[^ ^15^, ^27^ ^]^ Full datasets are available on zenodo (10.5281/zenodo.15130599).

##### ^13^C Solid‐State NMR Spectroscopy

All solid‐state NMR spectra were recorded on a Bruker Avance III HD 500WB spectrometer using double‐bearing MAS probes (DVT BL4) at resonance frequencies of 500.192 MHz and 125.773 MHz for ^1^H and ^13^C. The chemical shifts were referenced indirectly relative to an external TMS sample. All NMR spectra were recorded at ambient temperature (sensor temperature was 298 K). All spectra were collected in Topspin 3.6 and processed with MestreNova 15.0.0. Sample powders were finely ground with a mortar and pestle and were packed into a 4‐mm ZrO_2_ rotor from Bruker with a KelF or Vesp rotor cap. The amounts varied from 72.9 to 45.9 mg due to density differences between the samples. The ^13^C spectra were measured using CP‐MAS at a MAS rate of 10 kHz with 10 240 scans and a 6 s recycling delay (18 h). CP matching of 3 ms was achieved at 54 kHz by optimization of the ^1^H ramp pulse (90–100). ^1^ H‐decoupling was achieved with the tppm15 pulse train at 96 kHz. The ^13^C NMR spectra were analyzed to investigate the structure and cross‐linking properties of the organic RF xerogels. Using MNova software, peaks were assigned to specific structural motifs of the polymer framework. Peak deconvolution and integration were performed with ssNake software^[^ ^28^ ^]^ to quantify the contributions of individual signals, providing information on the degree of cross‐linking. CP‐MAS, which is inherently not strictly quantitative, was used as it is much more sensitive (then the alternative more quantitative direct polarization (DP)), so the experiments were significantly shorter, i.e., 18 h vs days for DP. The intensity of each peak is a function of the hydrogen density in the close vicinity. The latter should not vary much for each type of carbon across the different samples, allowing to monitor the evolution of each type of carbon during the reaction. The relative ratios between different types of carbons were not quantified. Full datasets are available on zenodo (10.5281/zenodo.15130599).

## Supporting Information

Supporting Information is available from the Wiley Online Library or from the author.

## Conflict of Interest

The authors declare no conflict of interest.

## Supporting information

Supplementary Material

## Data Availability

Data that are not provided here or in the supplementary information can be found on zenodo (10.5281/zenodo.15130599).
