# Supplementary material for: Comprehensive Analysis of Key Parameters Determining Formation and Structural Properties of Sol–Gel‐Derived Nanoporous Polymers
Source: Small Sci. 2025 Nov 18;6(1):e202500460. doi: 10.1002/smsc.202500460 (PMC12798777; doi:10.1002/smsc.202500460)
Supplement: Supplementary file 1 — Supplementary Material [file SMSC-6-e202500460-s001.pdf]

Supporting Information

Key Parameters Determining Formation and Structural Properties of Sol-Gel-Derived Nanoporous Polymers

*Abdurrahman Bilican, Priyanka Sharma, Glen J Smales, Markus Leutzsch, Christophe Farès, Heike Ehmann, Armin Moser, Claudia Weidenthaler, Wolfgang Schmidt **

In situ Small-angle X-ray Scattering

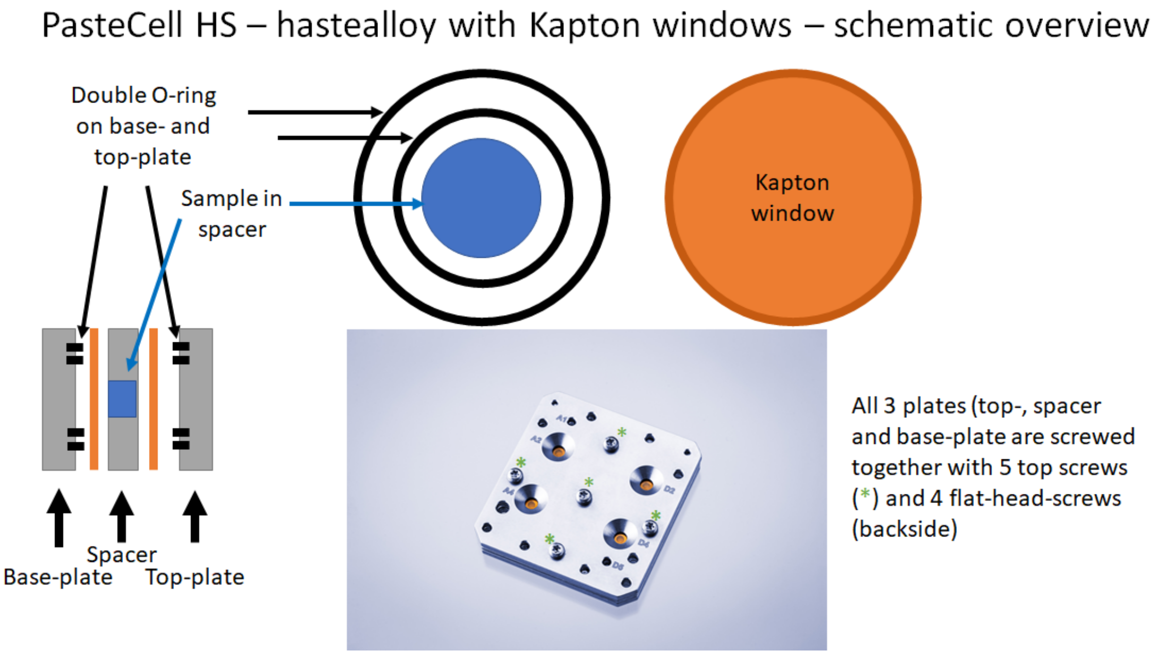

Scheme S1. Schematics of the reaction cell used for the im situ SAXS measurements.

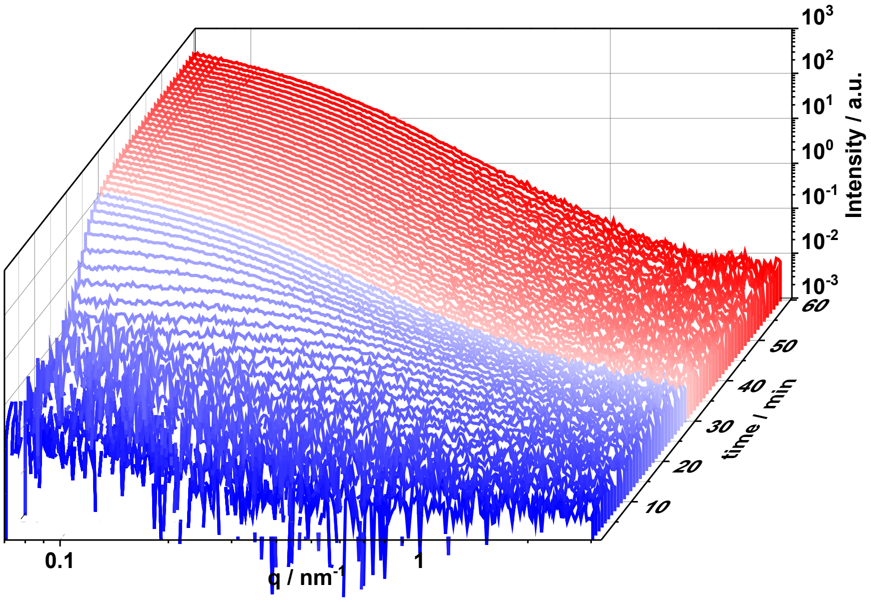

Figure S1. In-situ SAXS scattering curves of the gelation of RC 750/30 reaction solution at 80°C.

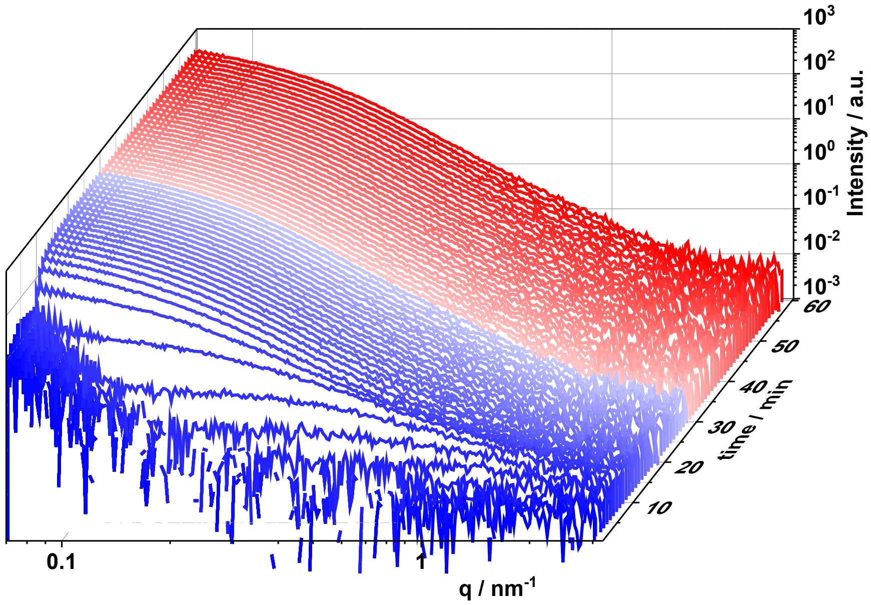

Figure S2. In-situ SAXS scattering curves of the gelation of RC 750/30 reaction solution at 100°C.

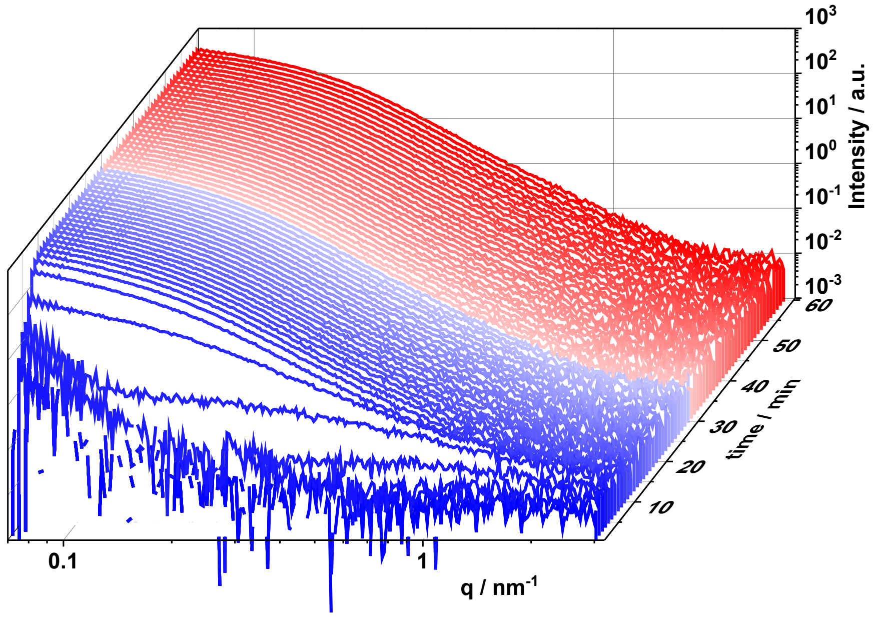

Figure S3. In-situ SAXS scattering curves of the gelation of RC 750/30 reaction solution at 120°C.

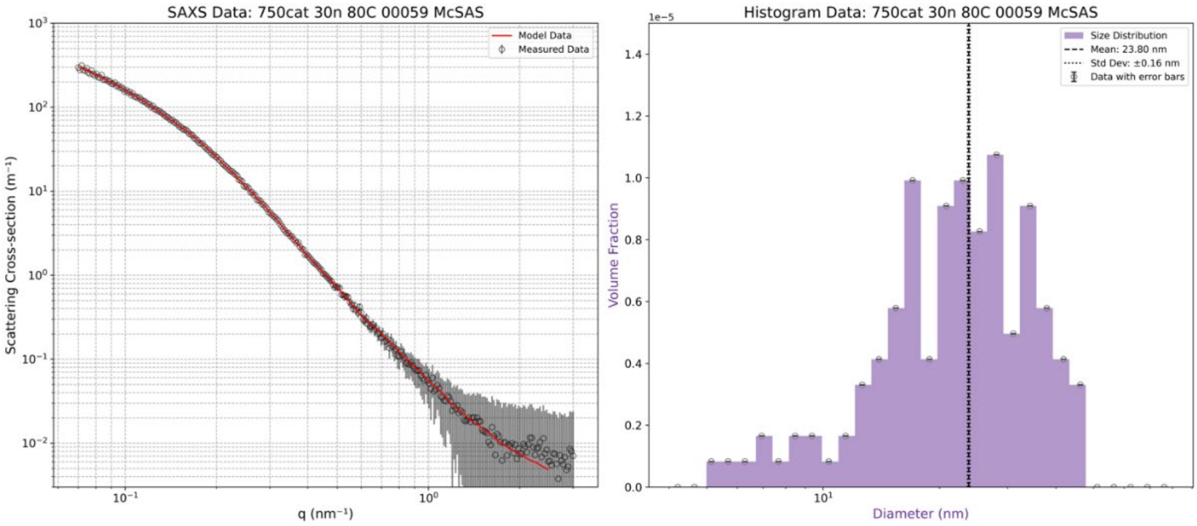

Figure S4. Fitted SAXS scattering curve (left, red) and the derived pore size distribution (right) of the RF xerogel after 60 min at 80°C.

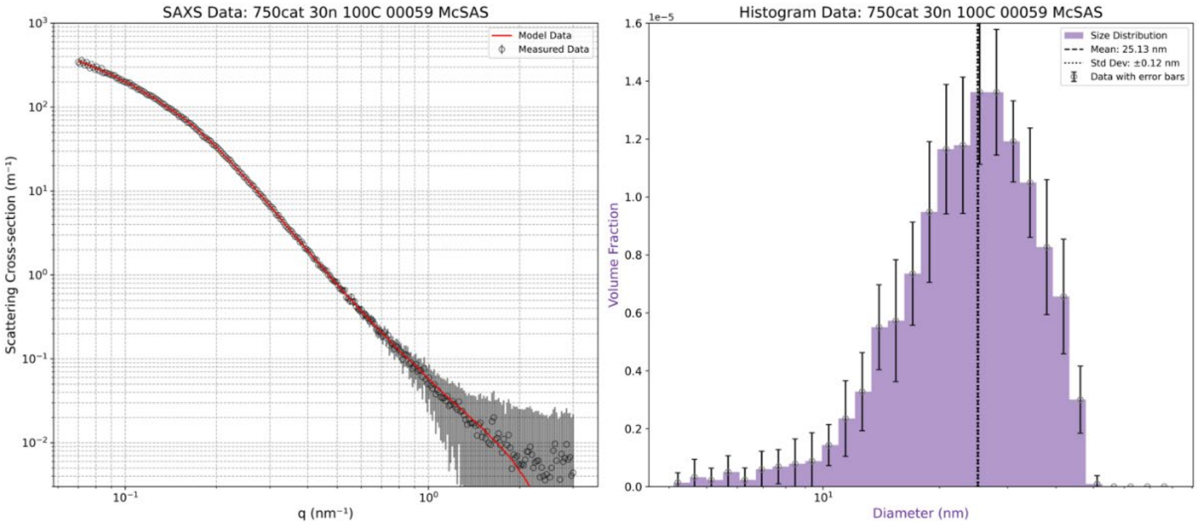

Figure S5. Fitted SAXS scattering curve (left, red) and the derived pore size distribution (right) of the RF xerogel after 60 min at 100°C.

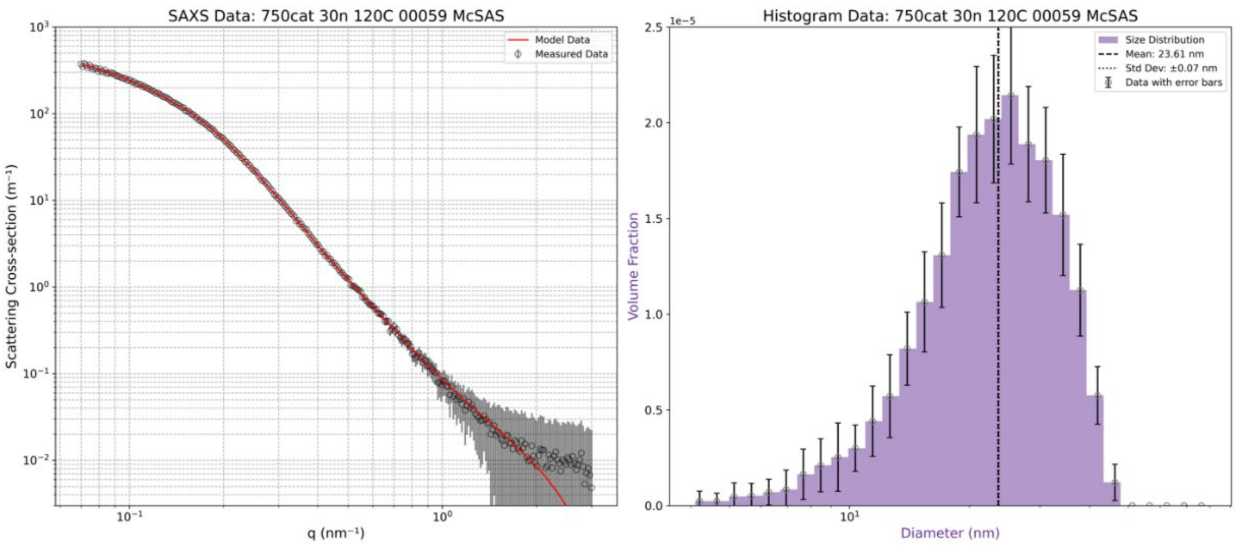

Figure S6. Fitted SAXS scattering curve (left, red) and the derived pore size distribution (right) of the RF xerogel after 60 min at 120°C.

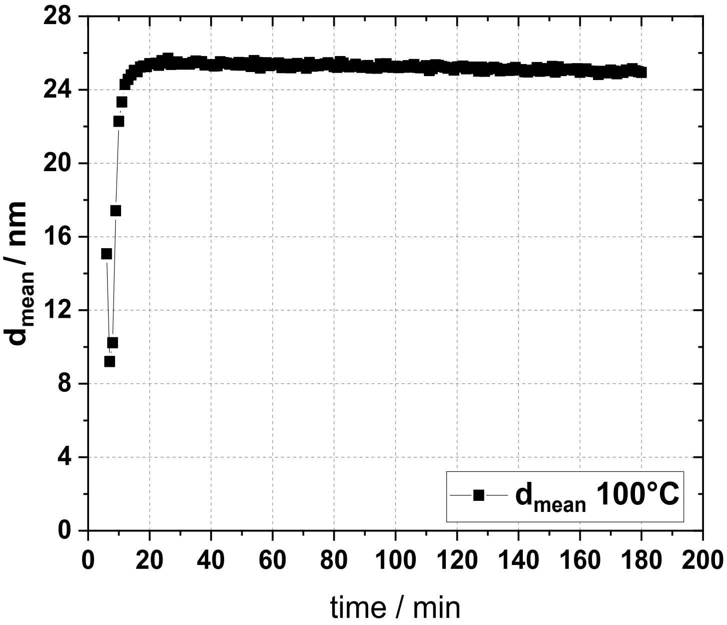

Figure S7. Mean pore size evolution derived from SAXS particle size distribution over 180 min at 100°C.

^{1}H NMR Spectroscopy

^{1}H NMR measurements allow tracking of the intensity of the characteristic meta-H triplet signal of resorcinol which can be used as an indicator for the reaction progress. When the reaction proceeds at other positions, this signal changes. The additional species in the ^{1}H NMR spectra were not analyzed in detail as the focus was on the consumption of resorcinol. When the reactionproceeds, the overall concentration of resorcinol-derived species in solution decreases due to the formation of a solidified crosslinked RF network. Resorcinol species in the solidified RF gel are not observable by standard solution-state NMR methods. Thus, the decrease in resorcinol concentration, as determined from the meta-H triplet signal, is a quantitative measure for the progress of the gel formation process.

A representative plot with the integrated signal of resorcinol molecules that remain dissolved in the reaction solution is shown in Figure S8.

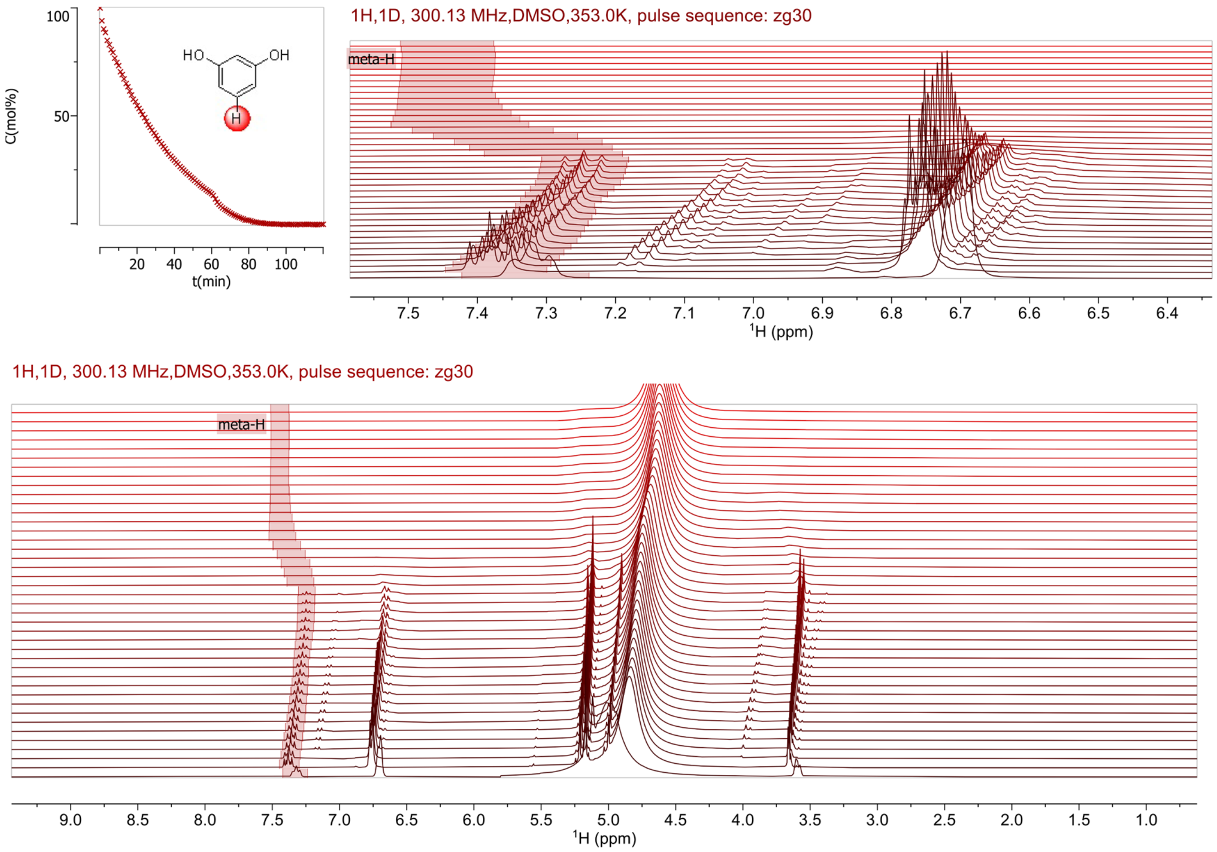

Figure S8. ^{1}H NMR spectra measured in situ during the gelation process of an RF gel at 80°C (bottom). At the top, the concentration decay of resorcinol, as calculated from the meta-H triplet signal, is shown (top left) as well as an enlarged image of the spectra (top right).

Cryoporometry

For the evaluation of the pore size distribution, the Gibbs-Thomson temperature decrease Δ T_m of the melting point in dependence to the crystallite size *x* and bulk melting point T_m described in Eq. (S1) is the physical basis of the analysis.[S1]

$$\Delta T_m = T_m - T_m(x) = \frac{4\gamma_{sl} T_m}{x \Delta H_f \rho_s} \tag{S5.1}$$

The surface energy at the solid-liquid interface γ_{sl}, and the bulk enthalpy of fusion ΔH_f, cannot be determined easily. Therefore, *Hansen et al.* proposed a simplification of Eq. (2.1.) The constant k_c and the surface layer thickness of the unfrozen pore water 2*sl*, can be determined empirically. Therefor the pore width values obtained from the BJH analysis can be used.[S2] According to Eq (2.2), a linear relationship where k_c and 2*sl* can be determined by linear fitting.[S3, S4]

$$\Delta T_m = \frac{k_c}{x - 2sl} \tag{S5.2}$$

$$x = \frac{k_c}{\Delta T_m} + 2sl \tag{S5.3}$$

The normalized pore size distribution d*v*/d*x* can be obtained from the first derivative d*v*/dT_M of the melting curve using Eq. 3.[S5]

$$\frac{dv}{dx} = \frac{k_c}{x^2} \cdot \frac{dv}{dT_m} \tag{S5.4}$$

Using the pore size distribution, the mode value of the pore width is obtained for hydrogels and xerogels, and the shrinkage is calculated using Eq. 5.

$$\varepsilon = \frac{x_{hydrogel} - x_{xerogel}}{x_{hydrogel}} \tag{S5.5}$$

Figure S9 shows melting curves from NMR cryoporometry of water within the pores of RF hydrogels obtained at 120 after 1 and 24 hours and within the pores of the corresponding dried RF xerogels.

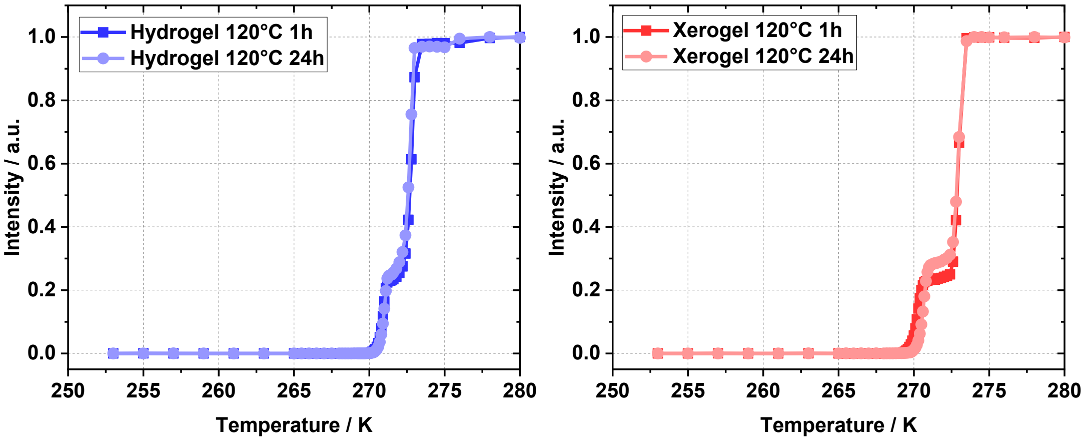

Figure S9. NMR melting curves of water impregnated in RF gels before (blue) and after drying (red).

^{13}C CP-MAS NMR Spectroscopy

^{13}C CP-MAS NMR spectroscopy was chosen for analysis for its excellent resolution and signal-to-noise ratio, although this method is only semi-quantitative. Care needs to be taken when comparing the intensity of signals. In this study, only the evolution of intensities (a.u.) of each individual peak were recorded for different samples under identical experimental conditions. The raw data obtained from the solid-state NMR experiments were processed systematically using the ssNake software to ensure accurate and consistent spectral analysis. Initially, the raw data were imported into the software, and the digital filter was corrected for. In order to reduce noise in the data, the FID was truncated to 256 points (about 2.6 ms, corresponding to the raw FID being completely attenuated) and then zero-filled to 1028. Next, the signal was subjected to apodization using a Lorentzian function with a width parameter of 100 Hz. The apodized time-domain data were then Fourier transformed to produce the NMR spectrum, which was phase-corrected manually. The pivot point was set at the midpoint of all peaks, and both zero-order and first-order phase correction parameters were adjusted until all relevant peaks exhibited an in-phase line-shape along a continuously curved baseline. To finalize the data treatment, a baseline correction was applied using a polynomial fit of order 5, effectively eliminating baseline distortions and ensuring a flat baseline for accurate peak integration and spectral analysis. This comprehensive data treatment approach allowed for the generation of high-quality NMR spectra with minimized noise. For each peak region (a to g, defined/assigned in Tables S1 and S2), a Lorentzian shape was defined in ssNake. The simulated peak position, intensity and line-shape (combined Gaussian and Lorentzian) were first adjusted manually and optimized by iterative fitting using a built-in simplex algorithm, with the goal to minimize the root-mean-square deviation (RMSD) between the experimental and simulated spectrum.

For illustration, Tables S1 and S2 report the integrated peak areas of the ^{13}C NMR resonances visible in the spectra shown in Figures S10 to S21.

Table S1. Assignment of ^{13}C resonances to carbons within the polymer network and their

multiplier			0.932	0.813	0.870	0.921	0.918	0.847
**kHz**	**ppm**	**assignment**	**0.5 h**	**1 h**	**2 h**	**4 h**	**8 h**	**24 h**
**18.99**	151.0	C_{aro}-OH (pos a)	1.91E+12	1.93E+12	1.85E+12	1.85E+12	1.83E+12	1.90E+12
**19.98**	158.9	C_{aro}-OH (pos a)	3.53E+10	3.75E+10	7.15E+10	6.67E+10	8.54E+10	7.73E+10
**16.99**	135.1	C_{aro}-H (pos b)	4.56E+10	8.51E+10	1.60E+11	1.40E+11	1.17E+11	4.13E+10
**16.39**	130.3	C_{aro}-H (pos b)	7.70E+11	7.69E+11	8.15E+11	7.95E+11	8.03E+11	7.44E+11
**14.95**	118.9	C_{aro}-CH_2 (pos c,d)	2.05E+12	2.08E+12	2.15E+12	2.15E+12	2.17E+12	2.12E+12
**14.28**	113.5	C_{aro}-CH_2 (pos c,d)	4.67E+11	5.57E+11	5.38E+11	5.61E+11	5.55E+11	6.38E+11
**12.99**	103.3	C_{aro}-H (pos e)	9.17E+10	5.02E+10	4.10E+10	3.70E+10	3.00E+10	6.00E+09
**3.584**	28.5	CH_2 (pos f,g)	9.27E+11	9.35E+11	1.03E+12	9.98E+11	9.84E+11	9.03E+11
**2.694**	21.4	CH_2 (pos f,g)	5.96E+11	5.94E+11	5.38E+11	6.15E+11	6.32E+11	6.68E+11

Integrals of the fitted peak areas obtained with ssNake for RF xerogels synthesized at 80°C.

Table S2. Assignment of ^{13}C resonances to carbons within the polymer network and their Integrals of the fitted peak areas obtained with ssNake for RF xerogels synthesized at 120°C.

multiplier			1.137	1.39	1.046	0.924	0.591	0.773
**kHz**	**ppm**	**assignment**	**0.5 h**	**1 h**	**2 h**	**4 h**	**8 h**	**24 h**
**19.02**	151.2	C_{aro}-OH (pos a)	1.98E+12	1.75E+12	2.00E+12	1.99E+12	1.99E+12	1.95E+12
**20.2**	160.6	C_{aro}-OH (pos a)	3.66E+10	4.59E+10	7.36E+10	8.37E+10	9.96E+10	1.40E+11
**16.99**	135.1	C_{aro}-H (pos b)	4.59E+10	1.11E+11	1.58E+11	1.85E+11	1.85E+11	2.03E+11
**16.44**	130.7	C_{aro}-H (pos b)	7.26E+11	6.68E+11	7.66E+11	7.78E+11	7.72E+11	7.76E+11
**14.99**	119.2	C_{aro}-CH_2 (pos c,d)	1.91E+12	1.65E+12	1.88E+12	1.88E+12	1.87E+12	1.91E+12
**14.3**	113.7	C_{aro}-CH_2 (pos c,d)	4.79E+11	5.53E+11	6.80E+11	6.95E+11	7.24E+11	7.35E+11
**12.98**	103.2	C_{aro}-H (pos e)	2.20E+11	1.20E+11	1.11E+11	1.10E+11	7.50E+10	6.07E+10
**3.63**	28.9	CH_2 (pos f,g)	1.00E+12	8.37E+11	9.85E+11	9.73E+11	9.91E+11	1.00E+12
**2.81**	22.3	CH_2 (pos f,g)	6.80E+11	7.28E+11	8.41E+11	8.47E+11	8.67E+11	8.46E+11

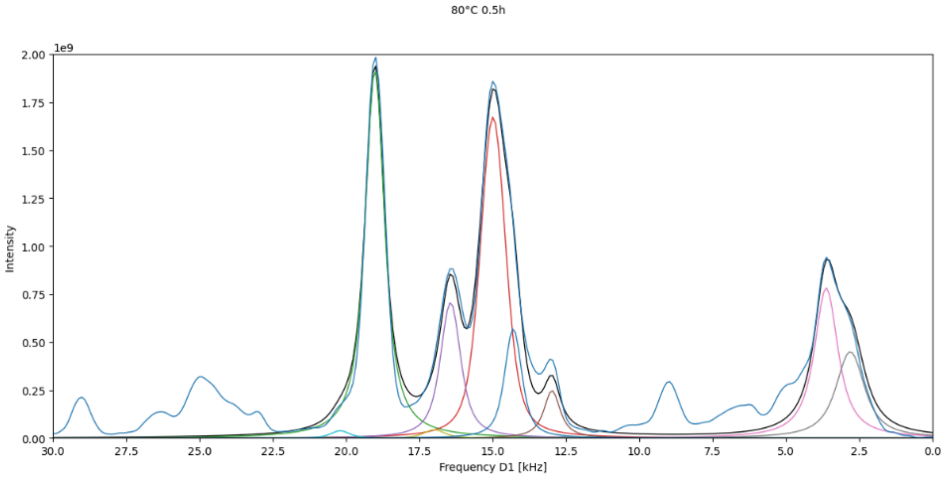

Figure S10. ^{13}C CPMAS NMR spectrum of RF xerogel synthesized at 80°C at 0.5 h.

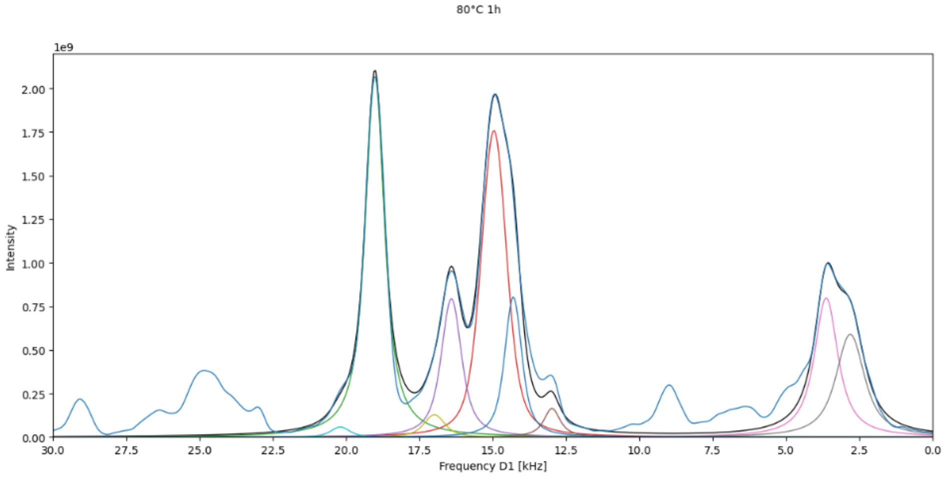

Figure S11. ^{13}C CPMAS NMR spectrum of RF xerogel synthesized at 80°C at 1 h.

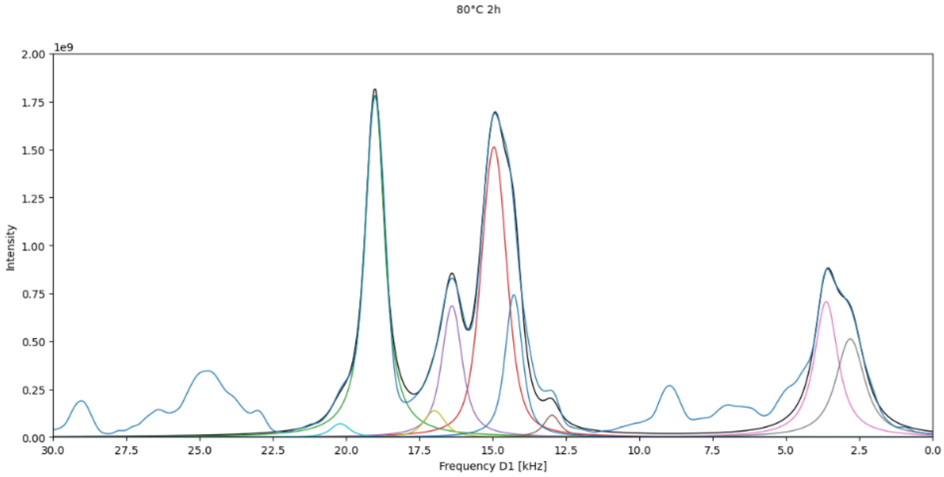

Figure S12. ^{13}C CPMAS NMR spectrum of RF xerogel synthesized at 80°C at 2 h.

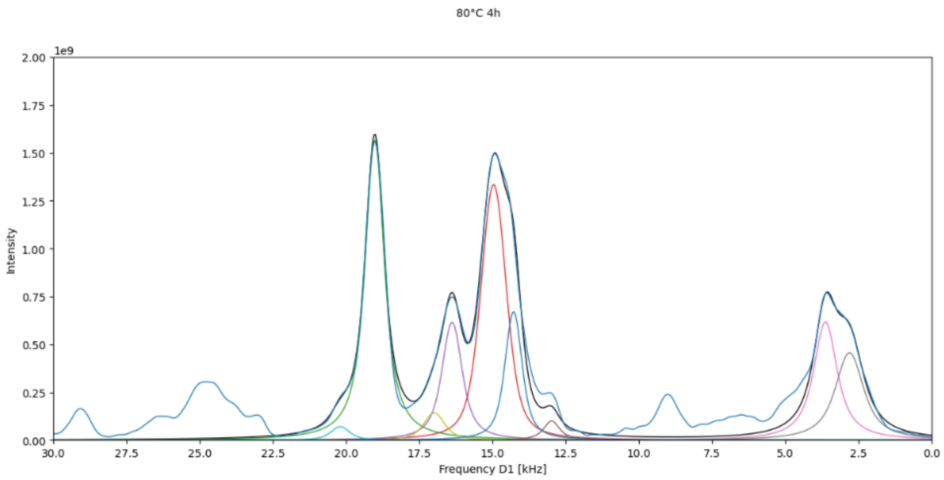

Figure S13. ^{13}C CPMAS NMR spectrum of RF xerogel synthesized at 80°C at 4 h.

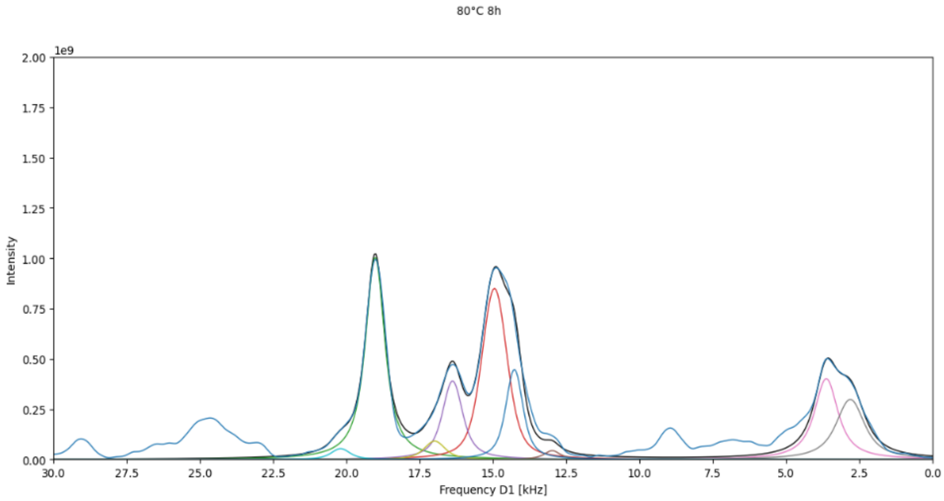

Figure S14. ^{13}C CPMAS NMR spectrum of RF xerogel synthesized at 80°C at 8 h.

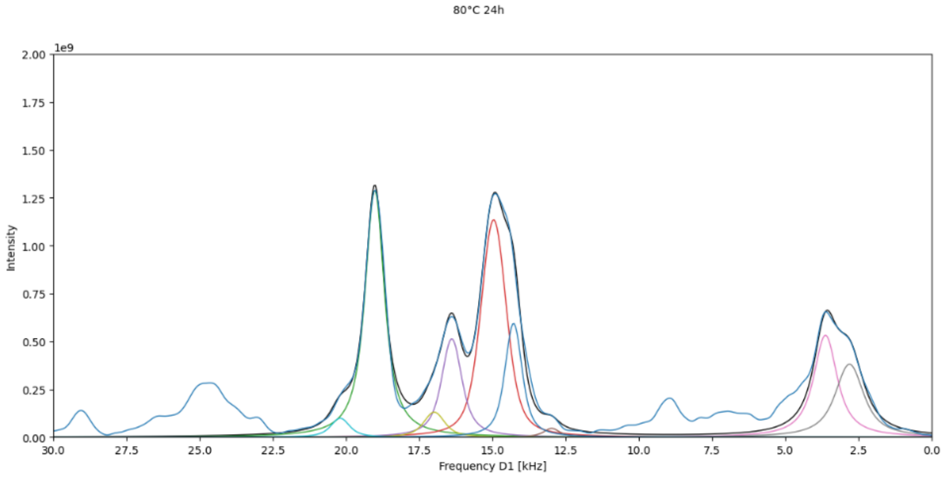

Figure S15. ^{13}C CPMAS NMR spectrum of RF xerogel synthesized at 80°C at 24 h.

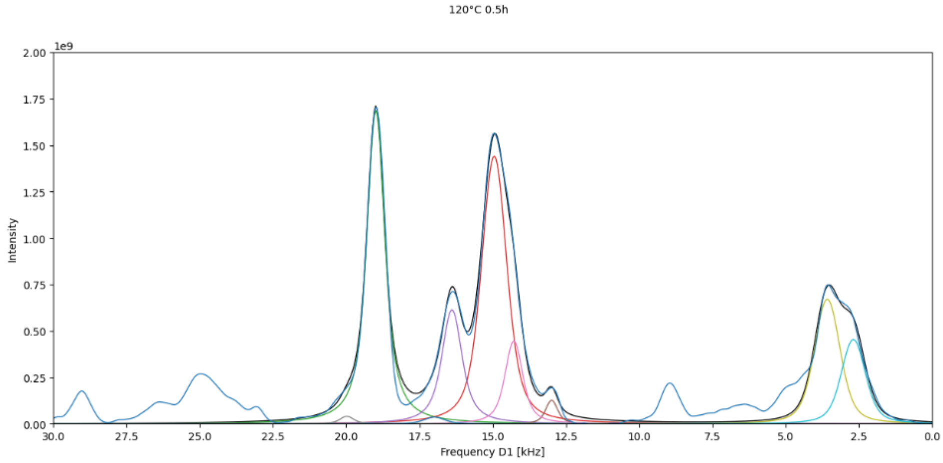

Figure S16. ^{13}C CPMAS NMR spectrum of RF xerogel synthesized at 120°C at 0.5 h.

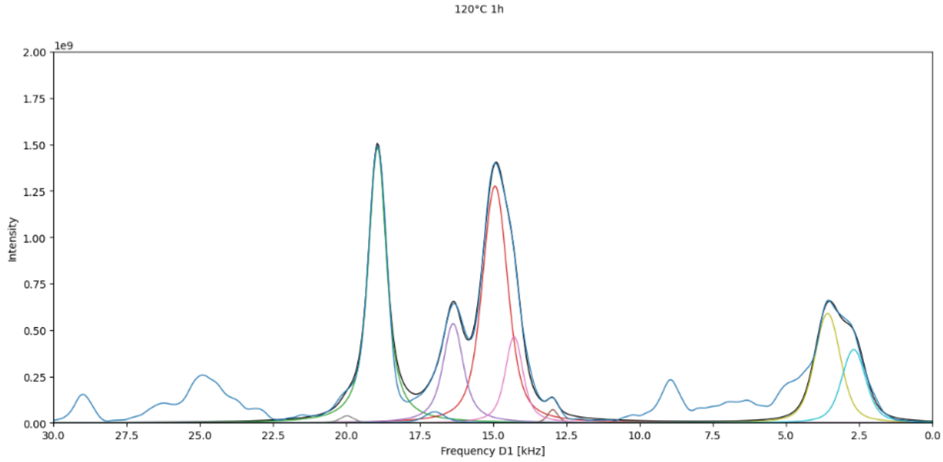

Figure S17. ^{13}C CPMAS NMR spectrum of RF xerogel synthesized at 120°C at 1 h.

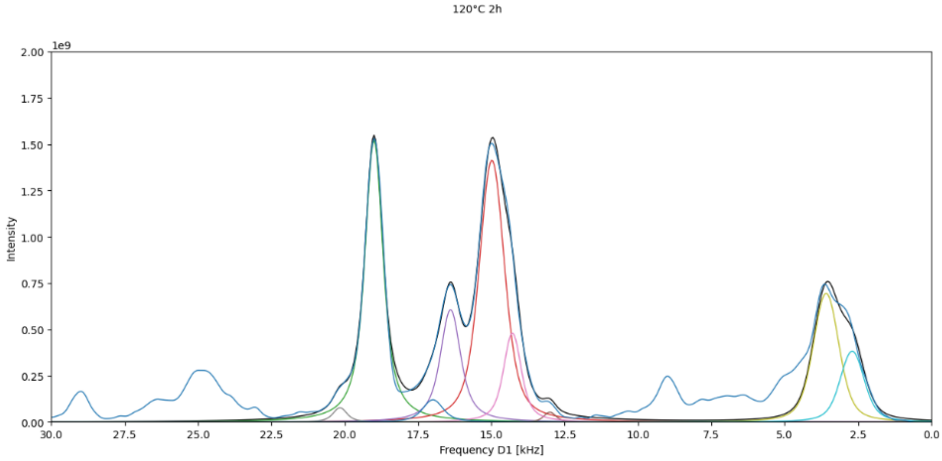

Figure S18. ^{13}C CPMAS NMR spectrum of RF aerogel synthesized at 120°C at 2 h.

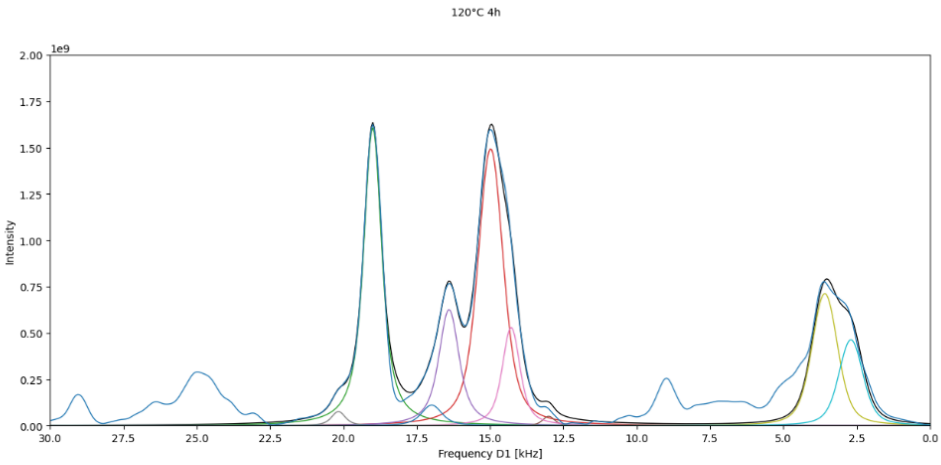

Figure S19. ^{13}C CPMAS NMR spectrum of RF aerogel synthesized at 120°C at 4 h.

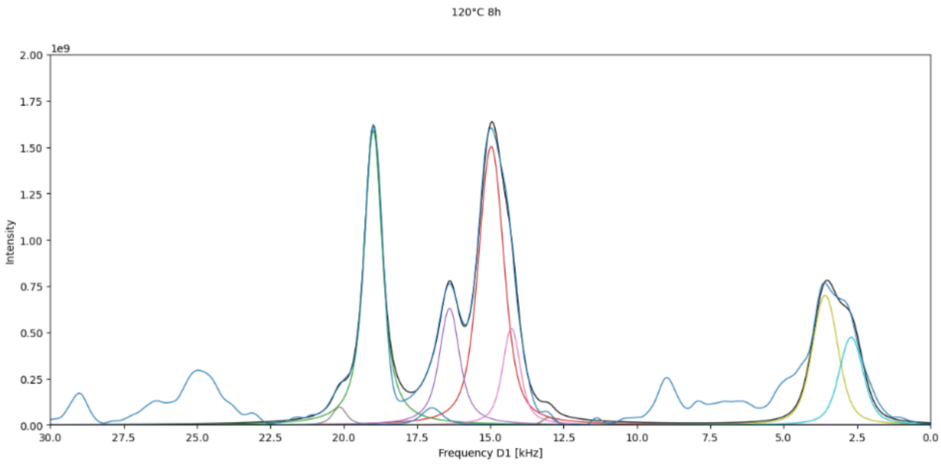

Figure S20. ^{13}C CPMAS NMR spectrum of RF aerogel synthesized at 120°C at 8 h.

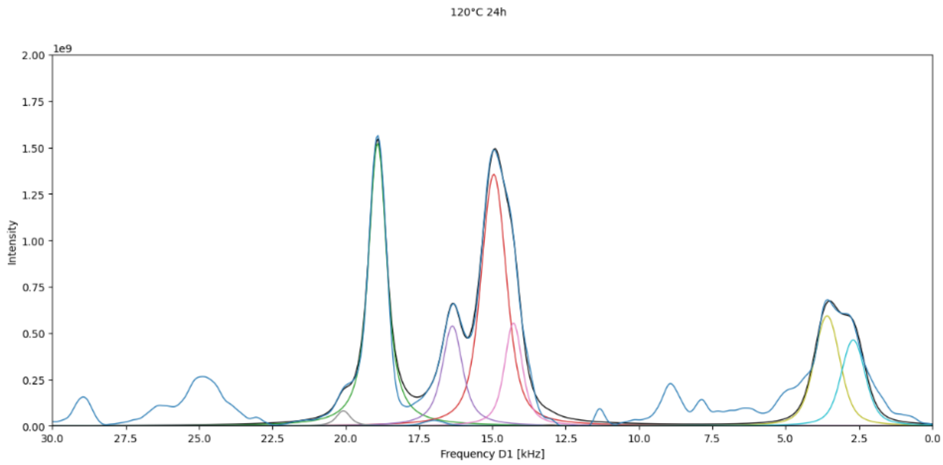

Figure S21. ^{13}C CPMAS NMR spectrum of RF xerogel synthesized at 120°C at 24 h.

Supplementary Literature:

S1. Jackson, C. L.; McKenna, G. B., The melting behavior of organic materials confined in porous solids. *The Journal of Chemical Physics* **1990,** *93* (12), 9002-9011.

S2. Hansen, E. W.; Schmidt, R.; Stöcker, M., Pore Structure Characterization of Porous Silica by 1H NMR Using Water, Benzene, and Cyclohexane as Probe Molecules. *The Journal of Physical Chemistry* **1996,** *100* (27), 11396-11401.

S3. Dore, J.; Webber, B.; Strange, J.; Farman, H.; Descamps, M.; Carpentier, L., Phase transformations for cyclohexane in mesoporous silicas. *Physica A: Statistical Mechanics and its Applications* **2004,** *333*, 10-16.

S4. Mitchell, J.; Webber, J. B. W.; Strange, J. H., Nuclear magnetic resonance cryoporometry. *Physics Reports* **2008,** *461* (1), 1-36.

S5. Strange, J. H.; Rahman, M.; Smith, E. G., Characterization of porous solids by NMR. *Physical Review Letters* **1993,** *71* (21), 3589-3591.
